# Triethyl­ammonium (indane-1,2,3-trione 1,2-dioximato-κ^2^ *N* ^1^,*O* ^2^)(indane-1,2,3-trione 2-oximato 1-oxime-κ^2^ *N* ^1^,*O* ^2^)nickel(II)

**DOI:** 10.1107/S1600536812010458

**Published:** 2012-03-14

**Authors:** Baoyun Zhong, Shengli Li, Guifang Chen

**Affiliations:** a The Third Middle School in Liaocheng, Shandong 252059, People’s Republic of China; b Department of Chemistry and Biology, Dongchang College Liaocheng University, Shandong 252059, People’s Republic of China

## Abstract

In the title compound, (C_6_H_16_N)[Ni(C_9_H_4_N_2_O_3_)(C_9_H_5_N_2_O_3_)], the Ni^II^ion is four-coordinated by two N atoms and two O atoms from two indane-1,2,3-trione-1,2-dioxime ligands. The two organic ligands are linked by an intra­molecular O—H⋯O hydrogen bond. In the crystal, mol­ecules are linked by N—H⋯O hydrogen-bonds.

## Related literature

For the use of oximes, see: Chaudhuri (2003 ▶). For theoretical research on their magnetic properties, see: Pavlishchuk *et al.* (2003 ▶). For a related structure, see: Chen *et al.* (2010 ▶). For the properties of related complexes, see: Davidson *et al.* (2007 ▶).
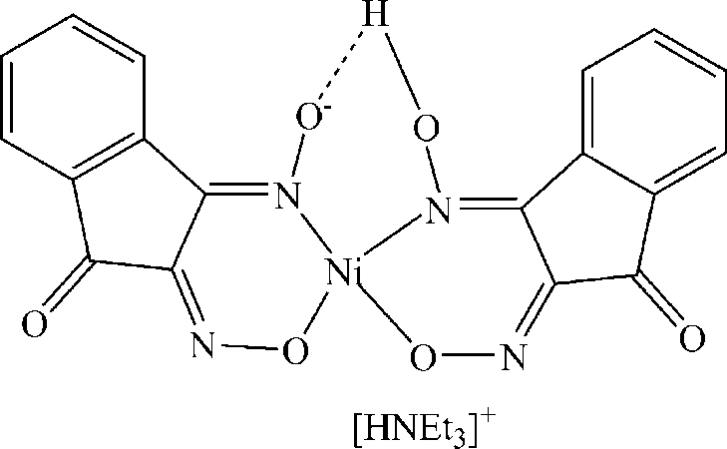


## Experimental

### 

#### Crystal data

(C_6_H_16_N)[Ni(C_9_H_4_N_2_O_3_)(C_9_H_5_N_2_O_3_)]
*M* *_r_* = 538.20
Triclinic, 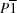

*a* = 9.710 (5) Å
*b* = 10.470 (5) Å
*c* = 12.156 (6) Å
α = 80.785 (6)°
β = 87.639 (6)°
γ = 72.217 (6)°
*V* = 1161.6 (10) Å^3^
*Z* = 2
Mo *K*α radiation
μ = 0.89 mm^−1^
*T* = 298 K
0.45 × 0.35 × 0.33 mm

#### Data collection

Siemens SMART CCD area-detector diffractometer
Absorption correction: multi-scan (*SADABS*; Sheldrick, 1996 ▶) *T*_min_ = 0.691, *T*_max_ = 0.758
6186 measured reflections
4073 independent reflections
2650 reflections with *I* > 2σ(*I*)
*R*_int_ = 0.027

#### Refinement

*R*[*F* ^2^ > 2σ(*F* ^2^)] = 0.044
*wR*(*F* ^2^) = 0.075
*S* = 1.00
4073 reflections
332 parameters
H atoms treated by a mixture of independent and constrained refinement
Δρ_max_ = 0.34 e Å^−3^
Δρ_min_ = −0.27 e Å^−3^

### 

Data collection: *SMART* (Siemens, 1996 ▶); cell refinement: *SAINT* (Siemens, 1996 ▶); data reduction: *SAINT*; program(s) used to solve structure: *SHELXS97* (Sheldrick, 2008 ▶); program(s) used to refine structure: *SHELXL97* (Sheldrick, 2008 ▶); molecular graphics: *SHELXTL* (Sheldrick, 2008 ▶); software used to prepare material for publication: *SHELXTL*.

## Supplementary Material

Crystal structure: contains datablock(s) I, global. DOI: 10.1107/S1600536812010458/vm2155sup1.cif

Structure factors: contains datablock(s) I. DOI: 10.1107/S1600536812010458/vm2155Isup2.hkl

Additional supplementary materials:  crystallographic information; 3D view; checkCIF report

## Figures and Tables

**Table 1:** Selected bond lengths (Å)

Ni1—O2	1.849 (2)
Ni1—N3	1.872 (3)
Ni1—O5	1.875 (2)
Ni1—N1	1.883 (2)

**Table 2:** Hydrogen-bond geometry (Å, °)

*D*—H⋯*A*	*D*—H	H⋯*A*	*D*⋯*A*	*D*—H⋯*A*
N5—H5⋯O5^i^	0.91	1.94	2.815 (3)	160
N5—H5⋯O2^i^	0.91	2.21	2.903 (3)	132
O4—H4⋯O1	1.18 (3)	1.19 (3)	2.363 (3)	177 (3)
O4—H4⋯N1	1.18 (3)	1.96 (3)	2.904 (3)	134 (2)

Symmetry code: (i) 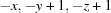.

**Fig. 1.**
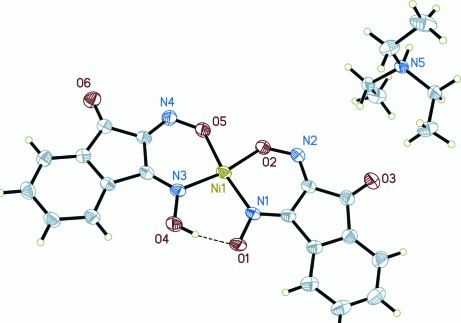
The molecular structure of the title compound with atom labels and 50% probability displacement ellipsoids for non-H atoms. Intramolecular hydrogen bond is shown as dashed line.

## supplementary crystallographic information

### Comment

There is currently a renewed interest in the coordination chemistry of oximes (Davidson *et al.*, 2007; Pavlishchuk *et al.*, 2003). The planar aromatic polyoxime ligand indane-1,2,3-trione-1,2-dioxime abbreviated as H_2_Itdo, was used for the synthesis of the title compound. The complex (Fig.1) consists of two indane-1,2,3-trione-1,2-dioxime ligands, Ni^2+^and one [HNEt_3_]^+^. The Ni center is four-coordinated by two N atoms and two O atoms from two indane-1,2,3-trione-1,2-dioxime ligands (Table 1). The sum of four angles around the Ni atom is 360.04 (1)° showing that the coordination is planar. There exists one deprotonated and one protonated oxime ligand with a strong intramolecular hydrogen bond between the OH group and the negatively charged oxygen of the other ligand (Table 2). The molecules are linked by weak C17—H17···O3^ii ^interactions (C17···O3 = 3.402 (5) Å, symmetry code: (ii) *x*+1, *y*, *z*+1) to give a 1D chain.

### Experimental

A solution of Ni(NO_3_)_2_ (0.0727 g, 0.25 mmol) in MeOH (10 ml) was added to a solution of indane-1,2,3-trione-1,2-dioxime (0.1056 g, 0.5 mmol) and 0.5 mmol NEt_3_ in MeOH (10 ml).The resulting black solution was stirred for about 6 h and was then allowed to slowly concentrate by solvent evaporation at room temperature. Green block crystals suitable for X-ray diffraction were obtained within one month (yield 30.6%, m.p. 310-320K).

### Refinement

All H atoms were placed in geometrically idealized positions (C—H 0.96 (methyl), C—H 0.97 (CH_2_), C—H 0.93 (phenyl) and N—H 0.91 Å) and treated as riding on their parent atoms, with *U*_iso_(H) = 1.2*U*_eq _or 1.5*U*_eq_(C), *U*_iso_(H) = 1.2*U*_eq_(O).

### Crystal data

**Table:**

(C_6_H_16_N)[Ni(C_9_H_4_N_2_O_3_)(C_9_H_5_N_2_O_3_)]	*Z* = 2
*M**_r_* = 538.20	*F*(000) = 560
Triclinic, *P*1	*D*_x_ = 1.539 Mg m^−^^3^
Hall symbol: -P 1	Mo *K*α radiation, λ = 0.71073 Å
*a* = 9.710 (5) Å	Cell parameters from 1572 reflections
*b* = 10.470 (5) Å	θ = 2.8–21.3°
*c* = 12.156 (6) Å	µ = 0.89 mm^−^^1^
α = 80.785 (6)°	*T* = 298 K
β = 87.639 (6)°	Block, green
γ = 72.217 (6)°	0.45 × 0.35 × 0.33 mm
*V* = 1161.6 (10) Å^3^	

### Data collection

**Table:**

Siemens SMART CCD area-detector diffractometer	4073 independent reflections
Radiation source: fine-focus sealed tube	2650 reflections with *I* > 2σ(*I*)
Graphite monochromator	*R*_int_ = 0.027
phi and ω scans	θ_max_ = 25.0°, θ_min_ = 2.5°
Absorption correction: multi-scan (*SADABS*; Sheldrick, 1996)	*h* = −7→11
*T*_min_ = 0.691, *T*_max_ = 0.758	*k* = −9→12
6186 measured reflections	*l* = −14→13

### Refinement

**Table:**

Refinement on *F*^2^	Primary atom site location: structure-invariant direct methods
Least-squares matrix: full	Secondary atom site location: difference Fourier map
*R*[*F*^2^ > 2σ(*F*^2^)] = 0.044	Hydrogen site location: inferred from neighbouring sites
*wR*(*F*^2^) = 0.075	H atoms treated by a mixture of independent and constrained refinement
*S* = 1.00	*w* = 1/[σ^2^(*F*_o_^2^) + (0.015*P*)^2^] where *P* = (*F*_o_^2^ + 2*F*_c_^2^)/3
4073 reflections	(Δ/σ)_max_ < 0.001
332 parameters	Δρ_max_ = 0.34 e Å^−^^3^
0 restraints	Δρ_min_ = −0.27 e Å^−^^3^

### Fractional atomic coordinates and isotropic or equivalent isotropic displacement parameters (Å^2^)

**Table:**

	*x*	*y*	*z*	*U*_iso_*/*U*_eq_	
Ni1	0.36828 (5)	0.15590 (4)	0.77300 (3)	0.04715 (15)	
N1	0.4326 (3)	0.0088 (2)	0.69308 (19)	0.0420 (7)	
N2	0.1501 (3)	0.2012 (3)	0.6025 (2)	0.0552 (8)	
N3	0.5272 (3)	0.1035 (2)	0.8702 (2)	0.0448 (7)	
N4	0.3108 (3)	0.3589 (3)	0.9226 (2)	0.0561 (8)	
N5	−0.0031 (3)	0.5196 (2)	0.24499 (19)	0.0427 (7)	
H5	−0.0878	0.5873	0.2293	0.051*	
O1	0.5575 (2)	−0.09090 (19)	0.71801 (18)	0.0532 (6)	
O2	0.2013 (2)	0.23611 (19)	0.68845 (17)	0.0577 (6)	
O3	0.0705 (3)	0.0957 (2)	0.41575 (18)	0.0704 (8)	
O4	0.6413 (2)	−0.0075 (2)	0.86314 (18)	0.0608 (7)	
H4	0.603 (3)	−0.051 (3)	0.791 (3)	0.078 (11)*	
O5	0.2738 (2)	0.3093 (2)	0.83908 (17)	0.0599 (7)	
O6	0.4210 (2)	0.4371 (2)	1.11162 (18)	0.0663 (7)	
C1	0.3587 (3)	−0.0071 (3)	0.6126 (2)	0.0413 (8)	
C2	0.2244 (4)	0.0886 (3)	0.5700 (2)	0.0459 (9)	
C3	0.1763 (4)	0.0403 (3)	0.4754 (3)	0.0530 (9)	
C4	0.2861 (4)	−0.0905 (3)	0.4650 (3)	0.0503 (9)	
C5	0.3935 (4)	−0.1196 (3)	0.5465 (2)	0.0458 (8)	
C6	0.5076 (4)	−0.2386 (3)	0.5533 (3)	0.0591 (10)	
H6	0.5798	−0.2600	0.6071	0.071*	
C7	0.5104 (4)	−0.3236 (3)	0.4778 (3)	0.0702 (11)	
H7	0.5856	−0.4045	0.4818	0.084*	
C8	0.4065 (5)	−0.2942 (4)	0.3965 (3)	0.0729 (12)	
H8	0.4136	−0.3535	0.3457	0.087*	
C9	0.2915 (4)	−0.1767 (4)	0.3900 (3)	0.0660 (11)	
H9	0.2195	−0.1565	0.3361	0.079*	
C10	0.5401 (3)	0.1702 (3)	0.9481 (2)	0.0417 (8)	
C11	0.4322 (3)	0.2923 (3)	0.9718 (3)	0.0444 (8)	
C12	0.4829 (4)	0.3399 (3)	1.0668 (3)	0.0472 (9)	
C13	0.6247 (3)	0.2408 (3)	1.0977 (2)	0.0418 (8)	
C14	0.6586 (3)	0.1395 (3)	1.0295 (2)	0.0413 (8)	
C15	0.7877 (3)	0.0347 (3)	1.0470 (2)	0.0508 (9)	
H15	0.8124	−0.0335	1.0027	0.061*	
C16	0.8782 (4)	0.0341 (3)	1.1312 (3)	0.0564 (9)	
H16	0.9649	−0.0357	1.1434	0.068*	
C17	0.8443 (4)	0.1341 (3)	1.1983 (3)	0.0579 (10)	
H17	0.9080	0.1317	1.2542	0.069*	
C18	0.7154 (4)	0.2375 (3)	1.1818 (3)	0.0528 (9)	
H18	0.6904	0.3043	1.2274	0.063*	
C19	0.0059 (4)	0.4224 (3)	0.1653 (2)	0.0528 (9)	
H19A	−0.0805	0.3938	0.1728	0.063*	
H19B	0.0069	0.4697	0.0900	0.063*	
C20	0.1351 (4)	0.2988 (3)	0.1811 (3)	0.0665 (11)	
H20A	0.2216	0.3256	0.1745	0.100*	
H20B	0.1345	0.2437	0.1253	0.100*	
H20C	0.1322	0.2477	0.2537	0.100*	
C21	−0.0122 (4)	0.4581 (3)	0.3641 (2)	0.0540 (10)	
H21A	0.0738	0.3814	0.3828	0.065*	
H21B	−0.0133	0.5247	0.4115	0.065*	
C22	−0.1422 (4)	0.4113 (3)	0.3886 (3)	0.0682 (11)	
H22A	−0.2266	0.4813	0.3576	0.102*	
H22B	−0.1528	0.3915	0.4678	0.102*	
H22C	−0.1308	0.3309	0.3563	0.102*	
C23	0.1134 (4)	0.5863 (3)	0.2317 (3)	0.0610 (10)	
H23A	0.2065	0.5175	0.2461	0.073*	
H23B	0.1003	0.6470	0.2864	0.073*	
C24	0.1133 (4)	0.6654 (3)	0.1169 (3)	0.0888 (14)	
H24A	0.1553	0.6039	0.0653	0.133*	
H24B	0.1687	0.7271	0.1177	0.133*	
H24C	0.0157	0.7158	0.0943	0.133*	

### Atomic displacement parameters (Å^2^)

**Table:**

	*U*^11^	*U*^22^	*U*^33^	*U*^12^	*U*^13^	*U*^23^
Ni1	0.0395 (3)	0.0407 (3)	0.0519 (3)	0.00054 (19)	−0.0017 (2)	−0.00464 (19)
N1	0.0343 (17)	0.0376 (15)	0.0459 (16)	−0.0028 (13)	0.0006 (13)	0.0007 (12)
N2	0.053 (2)	0.0496 (18)	0.0560 (18)	−0.0058 (15)	−0.0135 (15)	−0.0033 (15)
N3	0.0405 (18)	0.0402 (16)	0.0472 (16)	−0.0037 (13)	0.0045 (13)	−0.0059 (13)
N4	0.048 (2)	0.0550 (18)	0.0605 (19)	−0.0047 (15)	−0.0020 (15)	−0.0174 (15)
N5	0.0418 (18)	0.0368 (15)	0.0408 (15)	0.0009 (13)	−0.0014 (13)	−0.0056 (12)
O1	0.0382 (15)	0.0431 (13)	0.0675 (15)	0.0068 (11)	−0.0067 (12)	−0.0138 (12)
O2	0.0469 (16)	0.0497 (14)	0.0663 (15)	0.0053 (11)	−0.0157 (12)	−0.0144 (12)
O3	0.076 (2)	0.0643 (16)	0.0637 (16)	−0.0127 (14)	−0.0219 (14)	−0.0005 (13)
O4	0.0473 (16)	0.0564 (15)	0.0625 (15)	0.0146 (12)	−0.0102 (12)	−0.0191 (12)
O5	0.0477 (16)	0.0546 (14)	0.0645 (15)	0.0094 (11)	−0.0120 (12)	−0.0185 (12)
O6	0.0517 (17)	0.0601 (16)	0.0826 (17)	−0.0024 (13)	0.0047 (13)	−0.0284 (13)
C1	0.041 (2)	0.0376 (19)	0.0425 (19)	−0.0127 (16)	0.0075 (16)	0.0012 (15)
C2	0.045 (2)	0.0396 (19)	0.046 (2)	−0.0074 (17)	0.0006 (17)	0.0005 (16)
C3	0.065 (3)	0.049 (2)	0.044 (2)	−0.021 (2)	−0.0001 (19)	0.0026 (17)
C4	0.057 (3)	0.045 (2)	0.051 (2)	−0.0197 (19)	0.0041 (19)	−0.0045 (17)
C5	0.050 (2)	0.040 (2)	0.046 (2)	−0.0150 (17)	0.0103 (17)	−0.0019 (16)
C6	0.061 (3)	0.050 (2)	0.063 (2)	−0.0125 (19)	0.0074 (19)	−0.0091 (19)
C7	0.075 (3)	0.053 (2)	0.081 (3)	−0.014 (2)	0.018 (2)	−0.023 (2)
C8	0.101 (4)	0.059 (3)	0.066 (3)	−0.032 (3)	0.019 (2)	−0.025 (2)
C9	0.082 (3)	0.059 (2)	0.061 (2)	−0.026 (2)	−0.001 (2)	−0.010 (2)
C10	0.039 (2)	0.0417 (19)	0.0413 (19)	−0.0121 (16)	0.0048 (16)	0.0023 (15)
C11	0.030 (2)	0.0423 (19)	0.054 (2)	−0.0033 (16)	0.0000 (16)	−0.0046 (16)
C12	0.041 (2)	0.049 (2)	0.050 (2)	−0.0132 (18)	0.0045 (17)	−0.0035 (17)
C13	0.037 (2)	0.0432 (19)	0.047 (2)	−0.0165 (16)	0.0085 (16)	−0.0050 (16)
C14	0.037 (2)	0.0403 (19)	0.0421 (19)	−0.0099 (16)	0.0031 (16)	0.0019 (15)
C15	0.046 (2)	0.050 (2)	0.053 (2)	−0.0090 (17)	−0.0026 (18)	−0.0075 (17)
C16	0.043 (2)	0.052 (2)	0.066 (2)	−0.0035 (17)	−0.0082 (19)	−0.0031 (19)
C17	0.049 (3)	0.064 (2)	0.062 (2)	−0.022 (2)	−0.0099 (19)	−0.0009 (19)
C18	0.048 (2)	0.058 (2)	0.057 (2)	−0.0200 (19)	0.0050 (19)	−0.0147 (18)
C19	0.060 (2)	0.048 (2)	0.043 (2)	−0.0036 (18)	−0.0016 (17)	−0.0101 (16)
C20	0.063 (3)	0.050 (2)	0.083 (3)	−0.0069 (19)	0.021 (2)	−0.0266 (19)
C21	0.069 (3)	0.050 (2)	0.0364 (19)	−0.0085 (19)	−0.0028 (18)	−0.0075 (16)
C22	0.064 (3)	0.060 (2)	0.073 (2)	−0.013 (2)	0.020 (2)	−0.0048 (19)
C23	0.052 (3)	0.053 (2)	0.082 (3)	−0.0187 (19)	0.014 (2)	−0.020 (2)
C24	0.076 (3)	0.060 (2)	0.111 (3)	−0.012 (2)	0.038 (3)	0.014 (2)

### Geometric parameters (Å, º)

**Table:**

Ni1—O2	1.849 (2)	C9—H9	0.9300
Ni1—N3	1.872 (3)	C10—C11	1.446 (4)
Ni1—O5	1.875 (2)	C10—C14	1.473 (4)
Ni1—N1	1.883 (2)	C11—C12	1.485 (4)
N1—C1	1.298 (3)	C12—C13	1.469 (4)
N1—O1	1.345 (3)	C13—C18	1.366 (4)
N2—C2	1.294 (4)	C13—C14	1.402 (4)
N2—O2	1.322 (3)	C14—C15	1.388 (4)
N3—C10	1.295 (3)	C15—C16	1.374 (4)
N3—O4	1.350 (3)	C15—H15	0.9300
N4—C11	1.287 (3)	C16—C17	1.383 (4)
N4—O5	1.320 (3)	C16—H16	0.9300
N5—C23	1.491 (4)	C17—C18	1.379 (4)
N5—C19	1.496 (3)	C17—H17	0.9300
N5—C21	1.499 (3)	C18—H18	0.9300
N5—H5	0.9100	C19—C20	1.495 (4)
O1—H4	1.19 (3)	C19—H19A	0.9700
O3—C3	1.217 (4)	C19—H19B	0.9700
O4—H4	1.18 (3)	C20—H20A	0.9600
O6—C12	1.213 (3)	C20—H20B	0.9600
C1—C2	1.436 (4)	C20—H20C	0.9600
C1—C5	1.477 (4)	C21—C22	1.490 (4)
C2—C3	1.473 (4)	C21—H21A	0.9700
C3—C4	1.477 (4)	C21—H21B	0.9700
C4—C9	1.371 (4)	C22—H22A	0.9600
C4—C5	1.399 (4)	C22—H22B	0.9600
C5—C6	1.385 (4)	C22—H22C	0.9600
C6—C7	1.372 (4)	C23—C24	1.505 (4)
C6—H6	0.9300	C23—H23A	0.9700
C7—C8	1.373 (5)	C23—H23B	0.9700
C7—H7	0.9300	C24—H24A	0.9600
C8—C9	1.380 (4)	C24—H24B	0.9600
C8—H8	0.9300	C24—H24C	0.9600
			
O2—Ni1—N3	169.62 (10)	O6—C12—C13	126.9 (3)
O2—Ni1—O5	76.52 (9)	O6—C12—C11	128.3 (3)
N3—Ni1—O5	93.23 (10)	C13—C12—C11	104.8 (3)
O2—Ni1—N1	93.68 (10)	C18—C13—C14	121.3 (3)
N3—Ni1—N1	96.61 (11)	C18—C13—C12	128.1 (3)
O5—Ni1—N1	170.02 (11)	C14—C13—C12	110.6 (3)
C1—N1—O1	114.7 (2)	C15—C14—C13	119.3 (3)
C1—N1—Ni1	123.1 (2)	C15—C14—C10	132.2 (3)
O1—N1—Ni1	122.17 (19)	C13—C14—C10	108.5 (3)
C2—N2—O2	117.2 (3)	C16—C15—C14	118.5 (3)
C10—N3—O4	113.8 (2)	C16—C15—H15	120.7
C10—N3—Ni1	123.9 (2)	C14—C15—H15	120.7
O4—N3—Ni1	122.29 (19)	C15—C16—C17	122.0 (3)
C11—N4—O5	117.1 (3)	C15—C16—H16	119.0
C23—N5—C19	114.3 (2)	C17—C16—H16	119.0
C23—N5—C21	110.4 (2)	C18—C17—C16	119.5 (3)
C19—N5—C21	112.9 (2)	C18—C17—H17	120.2
C23—N5—H5	106.2	C16—C17—H17	120.2
C19—N5—H5	106.2	C13—C18—C17	119.3 (3)
C21—N5—H5	106.2	C13—C18—H18	120.3
N1—O1—H4	101.0 (14)	C17—C18—H18	120.3
N2—O2—Ni1	132.04 (17)	C20—C19—N5	114.8 (2)
N3—O4—H4	101.0 (15)	C20—C19—H19A	108.6
N4—O5—Ni1	131.86 (18)	N5—C19—H19A	108.6
N1—C1—C2	124.2 (3)	C20—C19—H19B	108.6
N1—C1—C5	128.6 (3)	N5—C19—H19B	108.6
C2—C1—C5	107.2 (3)	H19A—C19—H19B	107.6
N2—C2—C1	129.7 (3)	C19—C20—H20A	109.5
N2—C2—C3	121.4 (3)	C19—C20—H20B	109.5
C1—C2—C3	108.9 (3)	H20A—C20—H20B	109.5
O3—C3—C2	128.3 (3)	C19—C20—H20C	109.5
O3—C3—C4	126.1 (3)	H20A—C20—H20C	109.5
C2—C3—C4	105.6 (3)	H20B—C20—H20C	109.5
C9—C4—C5	121.5 (3)	C22—C21—N5	113.8 (3)
C9—C4—C3	128.8 (3)	C22—C21—H21A	108.8
C5—C4—C3	109.7 (3)	N5—C21—H21A	108.8
C6—C5—C4	119.8 (3)	C22—C21—H21B	108.8
C6—C5—C1	131.6 (3)	N5—C21—H21B	108.8
C4—C5—C1	108.6 (3)	H21A—C21—H21B	107.7
C7—C6—C5	117.6 (3)	C21—C22—H22A	109.5
C7—C6—H6	121.2	C21—C22—H22B	109.5
C5—C6—H6	121.2	H22A—C22—H22B	109.5
C6—C7—C8	122.6 (4)	C21—C22—H22C	109.5
C6—C7—H7	118.7	H22A—C22—H22C	109.5
C8—C7—H7	118.7	H22B—C22—H22C	109.5
C7—C8—C9	120.1 (3)	N5—C23—C24	112.1 (3)
C7—C8—H8	120.0	N5—C23—H23A	109.2
C9—C8—H8	120.0	C24—C23—H23A	109.2
C4—C9—C8	118.3 (3)	N5—C23—H23B	109.2
C4—C9—H9	120.9	C24—C23—H23B	109.2
C8—C9—H9	120.9	H23A—C23—H23B	107.9
N3—C10—C11	124.0 (3)	C23—C24—H24A	109.5
N3—C10—C14	129.0 (3)	C23—C24—H24B	109.5
C11—C10—C14	107.0 (3)	H24A—C24—H24B	109.5
N4—C11—C10	129.9 (3)	C23—C24—H24C	109.5
N4—C11—C12	121.1 (3)	H24A—C24—H24C	109.5
C10—C11—C12	109.1 (3)	H24B—C24—H24C	109.5
			
O2—Ni1—N1—C1	−2.3 (2)	C4—C5—C6—C7	0.3 (5)
N3—Ni1—N1—C1	179.0 (2)	C1—C5—C6—C7	−179.3 (3)
O5—Ni1—N1—C1	8.5 (7)	C5—C6—C7—C8	0.8 (5)
O2—Ni1—N1—O1	−180.0 (2)	C6—C7—C8—C9	−1.6 (6)
N3—Ni1—N1—O1	1.3 (2)	C5—C4—C9—C8	0.0 (5)
O5—Ni1—N1—O1	−169.2 (5)	C3—C4—C9—C8	179.7 (3)
O2—Ni1—N3—C10	8.2 (7)	C7—C8—C9—C4	1.2 (5)
O5—Ni1—N3—C10	−0.6 (3)	O4—N3—C10—C11	180.0 (2)
N1—Ni1—N3—C10	−179.0 (2)	Ni1—N3—C10—C11	1.3 (4)
O2—Ni1—N3—O4	−170.4 (5)	O4—N3—C10—C14	−1.6 (4)
O5—Ni1—N3—O4	−179.2 (2)	Ni1—N3—C10—C14	179.8 (2)
N1—Ni1—N3—O4	2.5 (2)	O5—N4—C11—C10	2.0 (5)
C2—N2—O2—Ni1	2.3 (4)	O5—N4—C11—C12	−179.8 (3)
N3—Ni1—O2—N2	172.0 (5)	N3—C10—C11—N4	−2.2 (5)
O5—Ni1—O2—N2	−179.0 (3)	C14—C10—C11—N4	179.1 (3)
N1—Ni1—O2—N2	−0.9 (3)	N3—C10—C11—C12	179.5 (3)
C11—N4—O5—Ni1	−1.4 (4)	C14—C10—C11—C12	0.8 (3)
O2—Ni1—O5—N4	−177.7 (3)	N4—C11—C12—O6	3.0 (5)
N3—Ni1—O5—N4	0.7 (3)	C10—C11—C12—O6	−178.5 (3)
N1—Ni1—O5—N4	171.3 (5)	N4—C11—C12—C13	−178.5 (3)
O1—N1—C1—C2	−178.1 (3)	C10—C11—C12—C13	0.0 (3)
Ni1—N1—C1—C2	4.1 (4)	O6—C12—C13—C18	−0.2 (6)
O1—N1—C1—C5	0.5 (4)	C11—C12—C13—C18	−178.7 (3)
Ni1—N1—C1—C5	−177.4 (2)	O6—C12—C13—C14	177.7 (3)
O2—N2—C2—C1	−0.8 (5)	C11—C12—C13—C14	−0.9 (3)
O2—N2—C2—C3	179.7 (3)	C18—C13—C14—C15	−0.8 (4)
N1—C1—C2—N2	−2.6 (5)	C12—C13—C14—C15	−178.9 (3)
C5—C1—C2—N2	178.6 (3)	C18—C13—C14—C10	179.4 (3)
N1—C1—C2—C3	177.0 (3)	C12—C13—C14—C10	1.4 (3)
C5—C1—C2—C3	−1.9 (3)	N3—C10—C14—C15	0.3 (5)
N2—C2—C3—O3	2.3 (6)	C11—C10—C14—C15	179.0 (3)
C1—C2—C3—O3	−177.4 (3)	N3—C10—C14—C13	−179.9 (3)
N2—C2—C3—C4	−179.0 (3)	C11—C10—C14—C13	−1.3 (3)
C1—C2—C3—C4	1.4 (3)	C13—C14—C15—C16	0.1 (4)
O3—C3—C4—C9	−1.3 (6)	C10—C14—C15—C16	179.8 (3)
C2—C3—C4—C9	179.9 (3)	C14—C15—C16—C17	0.0 (5)
O3—C3—C4—C5	178.4 (3)	C15—C16—C17—C18	0.6 (5)
C2—C3—C4—C5	−0.3 (4)	C14—C13—C18—C17	1.5 (5)
C9—C4—C5—C6	−0.7 (5)	C12—C13—C18—C17	179.2 (3)
C3—C4—C5—C6	179.5 (3)	C16—C17—C18—C13	−1.4 (5)
C9—C4—C5—C1	179.0 (3)	C23—N5—C19—C20	66.2 (3)
C3—C4—C5—C1	−0.8 (4)	C21—N5—C19—C20	−61.0 (4)
N1—C1—C5—C6	2.6 (6)	C23—N5—C21—C22	169.6 (3)
C2—C1—C5—C6	−178.7 (3)	C19—N5—C21—C22	−61.1 (3)
N1—C1—C5—C4	−177.1 (3)	C19—N5—C23—C24	59.2 (3)
C2—C1—C5—C4	1.7 (3)	C21—N5—C23—C24	−172.3 (2)

### Hydrogen-bond geometry (Å, º)

**Table:**

*D*—H···*A*	*D*—H	H···*A*	*D*···*A*	*D*—H···*A*
N5—H5···O5^i^	0.91	1.94	2.815 (3)	160
N5—H5···O2^i^	0.91	2.21	2.903 (3)	132
O4—H4···O1	1.18 (3)	1.19 (3)	2.363 (3)	177 (3)
O4—H4···N1	1.18 (3)	1.96 (3)	2.904 (3)	134 (2)

Symmetry code: (i) −*x*, −*y*+1, −*z*+1.

## References

[bb1] Chaudhuri, P. (2003). *Coord. Chem. Rev.* **243**, 143–190.

[bb2] Chen, Z. L., Jia, M. M., Zhang, Z. & Liang, F. P. (2010). *Cryst. Growth Des.* **10**, 4806-4814.

[bb3] Davidson, M. G., Johnson, A. L., Jones, M. D., Lunn, M. D. & Mahon, M. F. (2007). *Polyhedron*, **26**, 975–980.

[bb4] Pavlishchuk, V. V., Kolotilov, S. V., Addison, A. W., Prushan, M. J., Schollmeyer, D., Thompson, L. K., Weyhermuller, T. & Goreshnik, E. A. (2003). *Dalton Trans.* pp. 1587–1595.

[bb5] Sheldrick, G. M. (1996). *SADABS* University of Göttingen, Germany.

[bb6] Sheldrick, G. M. (2008). *Acta Cryst.* A**64**, 112–122. 10.1107/S0108767307043930 18156677

[bb7] Siemens (1996). *SMART* and *SAINT* Siemens Analytical X-ray Instruments Inc., Madison, Wisconsin, USA.

